# AR‐Enabled Persistent Human–Machine Interfaces via a Scalable Soft Electrode Array

**DOI:** 10.1002/advs.202305871

**Published:** 2023-12-13

**Authors:** Hodam Kim, Ho‐Seung Cha, Minseon Kim, Yoon Jae Lee, Hoon Yi, Sung Hoon Lee, Soltis Ira, Hojoong Kim, Chang‐Hwan Im, Woon‐Hong Yeo

**Affiliations:** ^1^ IEN Center for Human‐Centric Interfaces and Engineering Institute for Electronics and Nanotechnology Georgia Institute of Technology Atlanta GA 30332 USA; ^2^ George W. Woodruff School of Mechanical Engineering College of Engineering Georgia Institute of Technology Atlanta GA 30332 USA; ^3^ Department of Biomedical Engineering Hanyang University Seoul 04763 Republic of Korea; ^4^ School of Mechanical Engineering Soongsil University 369 Sangdo‐ro, Dongjak‐gu Seoul 06978 Republic of Korea; ^5^ School of Electrical and Computer Engineering College of Engineering Georgia Institute of Technology Atlanta GA 30332 USA; ^6^ Wallace H. Coulter Department of Biomedical Engineering College of Engineering Georiga Tech and Emory University School of Medicine Atlanta GA 30332 USA; ^7^ Parker H. Petit Institute for Bioengineering and Biosciences Institute for Materials Institute for Robotics and Intelligent Machines Neural Engineering Center Georgia Institute of Technology Atlanta GA 30332 USA

**Keywords:** augmented reality, electrode array, human–machine interface, soft wearable

## Abstract

Augmented reality (AR) is a computer graphics technique that creates a seamless interface between the real and virtual worlds. AR usage rapidly spreads across diverse areas, such as healthcare, education, and entertainment. Despite its immense potential, AR interface controls rely on an external joystick, a smartphone, or a fixed camera system susceptible to lighting. Here, an AR‐integrated soft wearable electronic system that detects the gestures of a subject for more intuitive, accurate, and direct control of external systems is introduced. Specifically, a soft, all‐in‐one wearable device includes a scalable electrode array and integrated wireless system to measure electromyograms for real‐time continuous recognition of hand gestures. An advanced machine learning algorithm embedded in the system enables the classification of ten different classes with an accuracy of 96.08%. Compared to the conventional rigid wearables, the multi‐channel soft wearable system offers an enhanced signal‐to‐noise ratio and consistency over multiple uses due to skin conformality. The demonstration of the AR‐integrated soft wearable system for drone control captures the potential of the platform technology to offer numerous human–machine interface opportunities for users to interact remotely with external hardware and software.

## Graphical abstract

This paper reports an AR‐integrated soft wearable electronic system that detects the gestures of a subject for more intuitive, accurate, and direct control of external systems. Specifically, the all‐in‐one wearable device includes a scalable electrode array and integrated wireless system to measure electromyograms for real‐time continuous recognition of hand gestures.

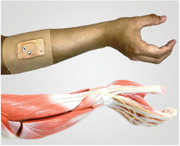

## 1 Introduction

Augmented reality (AR) is a computer graphics technology that establishes a harmonious blend of the real and virtual worlds. By skillfully integrating virtual elements and information into our physical surroundings, over 83 million users in the United States alone are projected to experience AR monthly by the end of 2023,^[^ ^1^ ^]^ interacting with diverse, layered digital enhancements over their real‐world surroundings. With a predicted market value of over $50 billion by 2024,^[^ ^1^ ^]^ AR adoption is exponentially flooding various sectors, including education, healthcare, entertainment, and retail. This trend shows the capability of AR to profoundly reshape societal interactions and human experiences. The embracement of AR, however, has encountered several challenges, primarily associated with its control interface usability. Typically reliant on hand‐held controllers,^[^ ^2^ ^]^ AR often imposes restrictive hand movements, making its usage limited and less user‐friendly. Despite the technological advancement in AR, the prevalent AR interfaces often entail the use of obtrusive hand‐held controllers, significantly limiting usability due to restrictive hand movements. This limitation is also prominent in camera‐based hand gesture recognition technologies,^[^ ^3^ ^]^ as in the case of AR headsets like the Microsoft HoloLens, and Oculus Quest, which demand constant visibility of the user's hands for gesture recognition, rendering them highly problematic for practical everyday use. This necessity, when paired with the technology's vulnerability to different lighting conditions, further diminishes the overall utility of AR interfaces. Here, a more user‐friendly, fully adaptable, and readily deployable AR solution that can overcome limitations and constraints in the current technology is needed.

Recent advances in real‐time analysis systems of electromyography (EMG), an electrical signal produced during muscle activity, and in conjunction with advancements in machine learning (ML) allow promising connection opportunities for enhancing the usability of AR. Since EMG contains insights into muscle activation and human muscle intention,^[^ ^4^ ^]^ wearable biosensors introduce a precise control system ensuring the precise classification of hand gestures. The forearm, an area abundant with muscles involved in hand movements, has been found advantageous for signal acquisition, leading to improved classification accuracy. Different gestures involve the activation of different muscles in the forearm. For example, flexing the wrist involves primarily the flexor muscles, while extending the wrist involves the extensor muscle. In addition, making a fist involves activating various forearm muscles working in harmony to flex the fingers and wrist. The activation and harmony of specific muscles will vary depending on the gesture, leading to distinct EMG signal patterns. Even in cases of amputees, EMG can be recorded from the remaining muscles in the forearm.^[^ ^5^ ^]^ To effectively classify hand gestures, measuring EMG from distinct specific muscles is crucial, playing a critical role in the classification process. Soft and skin‐like conformal sensors that can precisely measure these muscle signals provide a highly accurate system for classifying hand gestures,^[^ ^6^ ^]^ enabling a more sophisticated interaction with the AR environment. In contrast to computer vision‐based systems, decoding human intention with EMG overcomes the boundaries set by visual limitations. It allows for a more accurate interpretation of gestures, even in environments with varying light conditions. Unlike the light‐dependent nature of computer vision systems, EMG is immune to light fluctuations, making it an exceptionally robust tool that stands unrivaled in its ability to accurately detect and classify hand movements, reinforcing its unique advantage in gesture detection systems. Conversely, EMG signals have high individual variability caused by differences in physiological factors such as skin condition, muscle/fat mass, and structure of the neuromuscular system. Therefore, in terms of identifying hand/finger gestures, it is difficult to generalize the EMG signal pattern according to gesture and to apply the latest and state‐of‐the‐art technologies that require a lot of data for training, such as deep learning methods.

Here, we introduce an innovative solution combining EMG‐based soft bioelectronics with AR technology. Four primary areas are explored: 1) the development and validation of a soft, wireless forearm patch, emphasizing the flexibility and stretchability of versatile wearable applications; 2) the implementation of EMG‐based electronics to enable camera‐less hand gesture recognition, thereby overcoming limitations of AR hand tracking systems; 3) use of novel and effective ML techniques based on Riemannian feature that requires only short training of less than 1 min for real‐time hand gesture classification with an impressive average accuracy of 96.08%; and 4) establishing an AR application platform using the soft wearable device for both screen‐based working environment and complex teleoperation. The integration of these advanced technologies in wearable devices promises to propose new strides in bioelectronic devices and AR applications, offering a more intuitive, comfortable, and immersive user experience.

## 2 Result and Discussion

### 2.1 Overview of AR‐Enabled Persistent Human–Machine Interfaces via a Scalable Wireless Soft Electronic System

**Figure 1:**
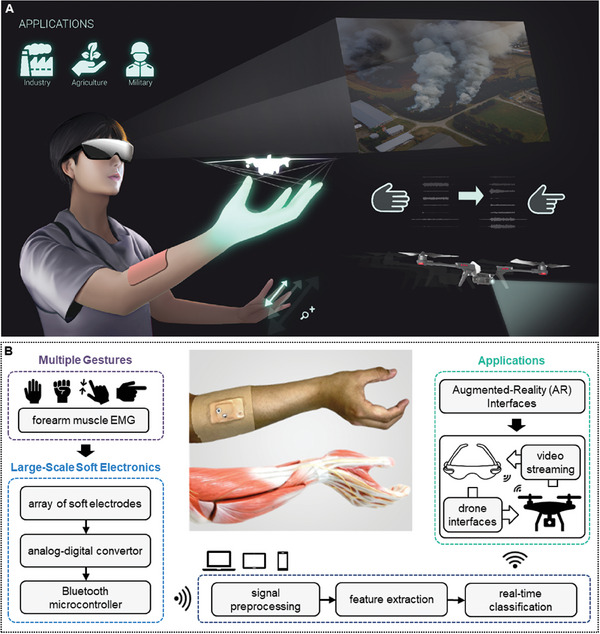
Overview of AR‐enabled persistent human–machine interfaces via a scalable wireless soft electronic system. A) An illustration of a subject wearing an AR headset and a soft electronic patch for drone control via hand/wrist gestures. B) A flow chart describing the step‐by‐step process, including EMG‐based gesture detection by using soft electronics, signal processing, data classification via machine learning, and real‐time control of a drone with AR interfaces. The photo in the middle shows a close‐up view of the soft electronic patch on the forearm and an illustration of forearm muscles where EMG signals are recorded through the device.

For the hand‐free interface of an AR system, we implement a scalable soft electronic device (SED) capable of recognizing hand/wrist gestures through EMG signals. **Figure** **1** overviews this innovative wireless SED‐based AR interface and a first‐person view (FPV) drone interface. A user wears AR glasses and puts the SED on the user's forearm to detect EMG signals. This setup enables the control of an AR virtual screen and drone actions through hand and wrist gestures. The AR‐integrated system allows users to control various virtual environments and external devices unrestrictedly in low‐light conditions, recognizing specific motions through the SED. Practical applications of this system span various fields, including industry,^[^ ^7^ ^]^ agriculture,^[^ ^8^ ^]^ and military.^[^ ^9^ ^]^ Figure 1B shows the overview of external device control using the SED, including gesture recognition, signal conversion, wireless data transfer, data classification, AR interface, and drone interface. The gesture recognition is enabled by EMG detection on the forearm, with the scalable SED having eight channels. The wearable device includes an analog‐digital converter, and a Bluetooth microcontroller, all working harmoniously for signal preprocessing and ML classification via a portable device. The AR interface is controlled by the measured EMG signals for real‐time, wireless, continuous control of an FPV drone with a video streaming capability.

### 2.2 Design, Architecture, and Fabrication of a Scalable Soft Wearable Device

**Figure 2:**
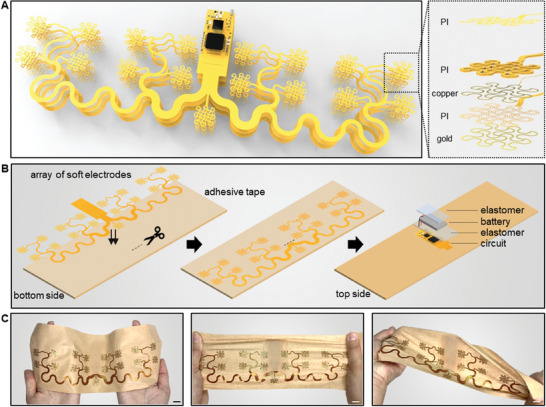
Design, architecture, and fabrication of a scalable soft wearable device. A) Illustration of a device, including an array of 17 electrodes (eight EMG channels), stretchable interconnector, and wireless circuit (left), along with a zoom‐in view of a single electrode structure composed of multiple layers of materials (right). B) Fabrication process, including the mounting of electrodes to the skin‐contact side of the adhesive tape, insertion of the circuit interconnects through a cut line to the top side of the tape, and the integration of chip components, a battery, and elastomer encapsulants. C) Photos showing the mechanical flexibility of the fabricated soft patch through bending, stretching, and twisting. Scale bars, 1 cm.

The presented SED integrates an array of flexible electrodes, stretchable interconnectors, and a wireless circuit onto a single flexible system (**Figure** **2**). This unique design offers a small form factor, skin conformality, and mechanical reliability to ensure user comfort, enhanced signal with minimal noise, and consistency over multiple uses. Incorporating all components into a single flexible board eliminates the need for additional parts and wiring, creating a more compact and lightweight system. This approach simplifies the manufacturing process and bolsters the system's overall reliability. One crucial advantage of using an all‐in‐one flexible board is its ability to maintain excellent signal integrity by minimizing soldered joints. This feature is particularly beneficial for applications dealing with high‐frequency signals or sensitive biopotential measurements, where maintaining the quality of the signal is of utmost importance. From a production standpoint, integrating all elements into one flexible board streamlines assembly, making manufacturing easier and more cost‐effective. Furthermore, the flexibility of the flexible board ensures it fits comfortably against the skin, reducing pressure points and preventing skin irritation. Figure 2A presents the schematic illustrations of an integrated electronic system, including an array of mesh electrodes, interconnectors, and a circuit. The flexible device provides enough flexibility to endure resistance change and stress concentration when placed on the forearm. The device size is 227.26 × 72.26 mm with a thickness of 0.5 mm. The wireless circuit consists of an analog‐to‐digital converter (ADC), microprocessor, antenna, and power regulator (Figure S1, Supporting Information). The ADC measures differential voltage from the electrodes and converts that into a digital signal. The microprocessor reads the digital signal and transmits it to an external device through a low‐power Bluetooth antenna (≈2.4 GHz). Serpentine‐patterned electrodes offer a conformal skin contact to lower the skin‐electrode contact impedance,^[^ ^10^ ^]^ increasing the effective contact area,^[^ ^11^ ^]^ and the skin‐electrode interfacial capacitance.^[^ ^12^ ^]^ As shown in Figure S2 (Supporting Information), we measured the skin‐electrode contact impedance of two electrode types: serpentine‐patterned and nonpatterned rectangular electrodes. When compared with the rectangular electrode, the serpentine electrode shows significantly lower impedance density (*p*‐value < 0.001). The forearm is one of the parts that get much movement as it performs various activities in our daily lives. Especially in agriculture, industry, and military, various shocks can be applied to the arm due to the user's actions or external stimuli, which can damage the SED when attached to the forearm. Therefore, we designed the electrode to enhance durability instead of maximizing flexibility and stretchability. Compared to the electrode described in the recent related work,^[^ ^13^ ^]^ our design is enhanced with intersecting serpentine patterns for reliability. This shows the system's endurance, ensuring that the overall signal measurement remains stable and reliable, even if an external stimulus damages part of an electrode. Recently, hydrogel‐based electrodes demonstrated excellent performance in conformal skin contact and mitigating motion artifacts.^[^ ^14^ ^]^ However, hydrogels' relatively low mechanical durability and temperature‐ or humidity‐sensitive nature often lead to poor performance in real‐life applications. On the contrary, the SED, consisting of a metal‐based dry electrode with high electrical conductivity, mechanical durability, and resistance to environmental changes, can be practically used in various environments without performance degradation. Figure 2B illustrates the device integration flow attaching the assembled circuit, interconnectors, and electrodes to the adhesive substrate: 1) cutting the adhesive substrates, 2) attaching interconnectors and electrodes to the adhesive layer by putting the assembled circuit part into the gap, 3) connecting a battery and encapsulation with elastomer. A lithium polymer battery (3.7 V, 150 mAh) with a slide switch and a circular magnetic recharging port is connected to the power regulator. Except for the switch and charging port, the overall circuit is encapsulated and soft‐packaged with an elastomer. The fabricated SED is bendable, stretchable, and twistable (Figure 2C). The wireless integrated device provides portable data acquisition without worrying about typical motion artifacts of multi‐channel wearable electrodes with a separate data acquisition system.^[^ ^15^ ^]^ In addition, the SED's flexibility ensures adaptation to body movements and changes, facilitating attachment to areas with frequent bending.^[^ ^16^ ^]^

### 2.3 Mechanical Characterization and Material Properties of the Soft Patch

**Figure 3:**
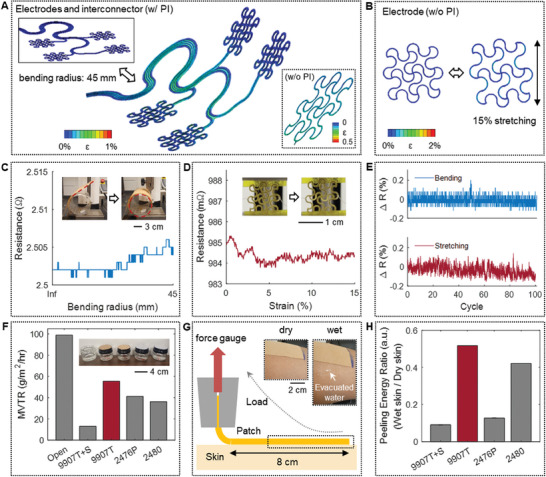
Mechanical characterization and material properties of the soft patch. A,B) Computational modeling results predicting mechanical stability during the bending of electrodes A) with interconnectors and B) the stretching of a single electrode. C) Measured electrical resistance of the interconnector and electrodes during the bending test (maximum bending radius: 45 mm). D) Electrical resistance of an electrode during stretching test (maximum strain: 15%) E) Change in electrical resistance throughout a cyclic bending test (100 cycles with 45 mm bending radius) and stretching tests (100 cycles with 15% strain) F) Experimentally measured MVTR values. The 9907T tape shows the highest breathability. G) A schematic illustration of an experiment for testing peeling energy. H) Peeling energy ratio between wet and dry skin for various adhesive substrates. The 9907T tape shows the most reliable adhesion in both dry and wet skin.

In this study, we used a finite element analysis tool (FEA; Abaqus, Dassault Systèmes) to design a flexible and stretchable mechanical structure. Continuous bending strain is applied when attaching a SED to the human's forearm. The average forearm circumference of adults ranges from ≈23 to 33 cm for males and 20 to 30 cm for females.^[^ ^17^ ^]^ Thus, the FEA results in **Figure** **3**A show the strain changes of the electrodes and interconnectors with a bending radius of 45 mm. The strain applied to the polyimide (PI) and copper layers is less than 1%. Additionally, the strain applied to the electrodes is less than 0.5%. In addition, the electrode design can endure stretching up to 15% with less than 2% of strain on the metal layer (Figure 3B). Based on the computational simulation results, we conducted a set of experimental validation studies. As shown in Figure 3C, the device shows negligible electrical resistance changes during the bending test (radius: 45 mm). The metal layer shows a resistance change of 4 mΩ, 0.16% of its original value. A stretching test in Figure 3D shows a negligible change in resistance with 1.1 mΩ, 0.11% of its original value. A cyclic bending and stretching test in Figure 3E further validates the device's mechanical stability with a negligible long‐term change before and after the test, a decrease of 5 mΩ (0.2%) and a decrease of 1.5 mΩ (0.15%), respectively. These small resistance changes cannot significantly affect the recording of electrophysiological signals.^[^ ^18^ ^]^ In addition, negligible resistance changes during continuous bending (3 mΩ; 0.12%) and stretching (0.9 mΩ; 0.09%) for 30 min show the signal stability provided by the SED (Figure S3, Supporting Information). Considering a continuous use case of the SED, breathability is important because moisture can affect the adhesion and the quality of the measured signal.^[^ ^13^, ^14^, ^19^ ^]^ Although a little sweat can increase the signal‐to‐noise ratio,^[^ ^19b^ ^]^ excessive sweating causes device delamination from the skin.^[^ ^20^ ^]^ Strong adhesion is essential to maintain consistent skin‐electrode contact impedance.^[^ ^21^ ^]^ The experimental setup in Figure 3F shows the results of calculating the moisture vapor transmission rate (MVTR). In this test, glass bottles containing 20 g of water were prepared, and the openings were covered with adhesive substrates. Four adhesive substrates were selected as candidates based on the literature survey.^[^ ^13^, ^16^, ^19^ ^]^ The MVTR was calculated by measuring water evaporation weight through each substrate for 24 h under room temperature conditions. As a result, the 9907T tape shows the highest MVTR (55.4877 g m^−2^ h) when compared to other adhesive tapes. An additional test includes a peeling force measure of substrates from the skin (Figure 3G), considering both dry and wet skin conditions (details in Figure S4A, Supporting Information). The result in Figure 3H shows that the 9907T tape shows the highest peeling energy ratio among the four candidates. The other three substrates had higher peeling energy in dry skin conditions (Figure S4B,C, Supporting Information). However, the adhesion was not maintained before the water was dropped on the skin. Considering both MVTR and peeling energy results, the 9907T tape was selected as the adhesive substrate to integrate the fabricated SED. Figure S5 (Supporting Information) shows the skin condition while wearing the SED for 8 h, and no side effects were observed, such as skin irritation and redness. This result is likely due to the synergy between wearing the SED using adhesion without additional pressure and good breathability.

### 2.4 Performance Validation of a Scalable Soft Wearable Device

**Figure 4:**
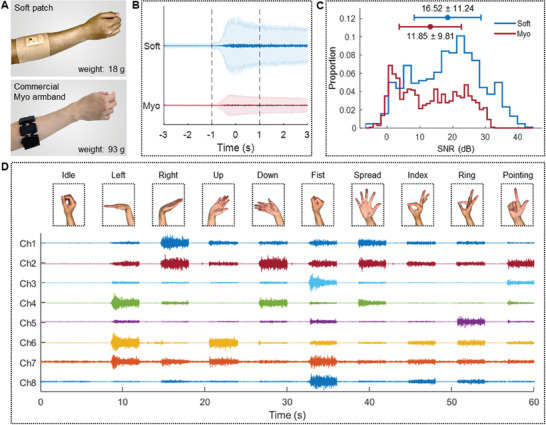
Performance validation of a scalable soft wearable patch. A) Photos of a soft patch and commercial armband (Myo) on the forearm for recording EMG during different gestures. B) Mean and standard deviation of EMG signals for ten hand gestures. C) Comparison of signal‐to‐noise ratio (SNR) of two devices, including a soft patch: 16.52 ± 11.24 and a commercial armband (Myo): 11.85 ± 9.81. D) Representative EMG signals measured by the soft wearable device with eight channels when a subject makes ten different gestures.

**Figure** **4**A shows two devices: one is the SED, and the other is a commercial armband‐type device (Myo). Both devices are mounted to the forearm for performance comparison and validation. The biggest difference is the weight and form factor. The patch‐type SED makes intimate contact with the skin without additional fixtures, while the rigid and heavy armband requires a tightening spring to secure the contact of rigid metal electrodes to the skin, causing discomfort and motion limits. For quantitative signal comparison, we developed a program that records EMG signals and presents hand gesture images (Figure S6, Supporting Information). A graph in Figure 4B shows the average and standard deviation of EMG signals measured from eight channels using ten hand gestures. After 3 s of resting with an idle gesture, a subject followed the instructions from the gesture detection program for 3 s. In the SNR calculation, the signal from −3 to −1 s was used as noise, and this signal from 1 to 3 s was used as the gesture signal. The SED and the commercial device cannot be sat in the same position on the arm because the electrode sizes and spacing between the pair electrodes of the two systems are all different. However, the EMG signals generated by activating specific forearm muscles can be conducted to the surroundings.^[^ ^22^ ^]^ The EMG signals recorded by the two devices show similar patterns, as shown in Figure S7 (Supporting Information). Therefore, we confirm that these devices sat near the same muscle groups on the forearm. Figure 4C presents the histogram of the calculated SNR values. The histogram distribution of SNR from the two devices compares the signal acquisition performance for various muscle groups and gestures. The SNR values of the soft patch and the device are 16.52 ± 11.24 and 11.85 ± 9.81 dB, respectively. The SNR of our patch was significantly higher than that of the commercial device (*p*‐value < 0.001). The SNR of SED was even higher than that of the commercial device under the wet skin condition (Figure S8, Supporting Information). The enhanced signal quality of the soft patch comes from skin conformality and natural motion detection by the lightweight, flexible device on the skin.^[^ ^23^ ^]^ Overall, the SED has eight channels to cover multiple muscles for more accurate differentiation of different gestures. An example in Figure 4D shows representative images and corresponding EMG signals from ten gestures made by a subject wearing the SED.

### 2.5 Signal Processing and Data Classification Methods

**Figure 5:**
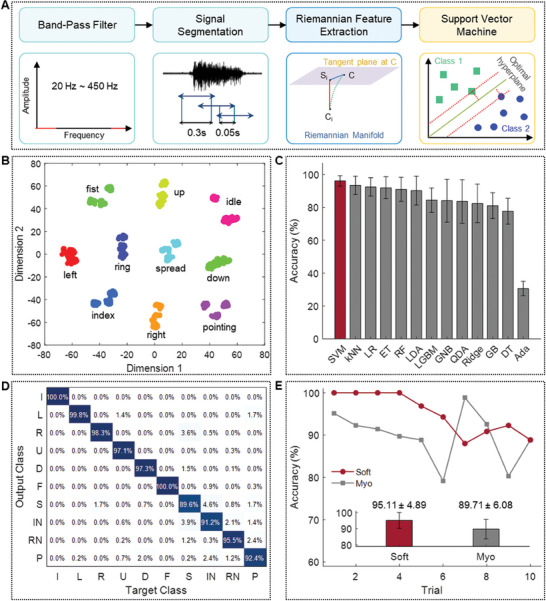
Signal processing and data classification methods. A) Signal processing of EMG for recognizing ten gestures. B) Distribution of extracted Riemann features representing ten gestures. C) Comparison of classification accuracies from 13 classifiers (SVM: support vector machine; kNN: k‐nearest neighbors; LR: logistic regression; ET: extra trees; RF: random forest; LDA: linear discriminant analysis; LGBM: light gradient boosting machine; GNB: gaussian naive bayes; QDA: quadratic discriminant analysis; Ridge: ridge regression; GB: gradient boosting; DT: decision tree; Ada: adaptive boosting). D) Confusion matrix showing an overall accuracy of 96.08% with ten gestures (I: idle; L: left; R: right; U: up; D: down; F: fist; S: spread; IN: index; RN: ring; P: pointing). E) Comparison of gesture recognition accuracy between a soft wearable patch and a commercial device (Myo). Among the total of 11 consecutive trials, the first trial is used as training data.

In this study, we developed a novel classification algorithm that requires short training time by combining recent feature extraction and machine‐learning methods to recognize hand/wrist gestures. **Figure** **5**A depicts the flow chart of the classification procedure. First, EMG signals recorded at all eight channels with a sampling rate of 2000 Hz are filtered using fourth‐order Butterworth band‐pass filters to remove 60 Hz power line noise and its harmonic frequency components. The cutoff frequencies of each filter are set to ± 8 Hz of the target. Then, the signals are further processed using a fourth‐order Butterworth bandpass filter with 20 and 450 Hz cutoff frequencies.^[^ ^22^, ^24^ ^]^ The filtered EMG signals are divided into short segments using a 300‐ms sliding window (600 samples) with 96% overlap. For each segment, a spatial covariance matrix (SCM) was extracted. The SCM is calculated by *SCM*  =  *XX^T^*/(*N* − 1), where *X* ∈ ℜ^C × N^is EMG signals with C the number of channels and N the number of time samples. The SCM is symmetric and positive‐definite matrix and can be regarded as a point on Riemannian manifolds. Then, the Riemannian average matrices were computed using SCMs, and the average matrices were mapped onto the Riemannian tangent space. The SCMs mapped onto the tangent space formed by the average matrices were used as the Riemannian feature. This tangent space mapping process allows the matrices to be vectorized and dealt with like Euclidian objects. In addition, this mapping processing allows the use of advanced classifiers available only in Euclidean space within the Riemannian space.^[^ ^25^ ^]^ All signal preprocessing and Riemannian feature extraction were performed using Python 3.8 and pyRiemann toolbox. Figure 5B shows the representation of our Riemannian features using t‐distributed stochastic neighbor embedding visualization.^[^ ^26^ ^]^ We confirm that all gestures are well clustered. Based on these well‐distributed Riemannian features extracted from EMG signals, ten hand/wrist gestures are classified using the ML method. A dataset consists of three trials, and each trial consists of ten hand/wrist gestures. In each cross‐validation, one trial is selected as the test dataset, and the remaining two trials are used as the training dataset. The Riemannian average matrices are computed using only the training dataset. Classification accuracy is calculated for each dataset, and a total of 15 datasets have been used to compare the classification performance of ten classifiers. As summarized in Figure 5C, the support‐vector machine (SVM) shows the highest classification accuracy of 96.08 ± 3.15%, and the confusion matrix of the classification outcome has resulted in Figure 5D. The hyperparameters of SVM were optimized using the grid search method for total datasets, and the classification accuracy of individual datasets was shown in Figure S9 (Supporting Information). SVM's optimized cost and gamma values were 1.09 and 1/72, respectively. Among ten gestures, the classification accuracy of the “spread” gesture was lower than that of other gestures, with a precision of 89.6%. This was because it was difficult to accurately perform the “spread” gesture. The “spread” gesture was misclassified as “right” and “index” gestures. Spreading the hand and bending the wrist are conducted by activating the extensor carpi ulnaris, extensor carpi radialis longus, and extensor carpi radialis brevis muscles. Excessive and forceful hand spreading and bending the wrist outward can produce a similar EMG signal when making a spread gesture. Similarly, in both “spread” and “index,” the middle, ring, and little finger are stretched commonly. In conclusion, the intricacies of overlapping muscle activations and EMG signal similarities are recognized as areas for enhancement. We acknowledge this complexity and are advancing our algorithm as a next step, enhancing the system's reliability and robustness for better practical applications. Our classification algorithm registers a performance that is slightly lower (by ≈1%) than some state‐of‐the‐art technologies utilizing deep learning methods for gesture recognition.^[^ ^27^ ^]^ In this study, our objective was to create a gesture recognition system that can be instantly usable upon wearing the SED. Therefore, some deep learning methods were unsuitable due to their extensive data and longer training time requirements. We optimized gesture recognition performance by integrating a novel Riemannian feature with SVM, requiring only a 1‐min training period. This is a significant improvement over the conventional methods that require tens or hundreds of sample data for each gesture.^[^ ^27^ ^]^ The minimal data requirement and reduced computational load of our algorithm enhance its practicality for real‐time applications and seamless integration into wearable and portable devices. Furthermore, SVM is suitable for real‐time classification due to less computational load and can be easily integrated into wearable and portable devices.

For the practical use of interfaces, minimizing the training time and the amount of training data required is important in maintaining performance with repeated use.^[^ ^28^ ^]^ We tested how long the classification accuracy is maintained with just one‐time training. For this long‐term usability test, we conducted an additional experiment in which ten gestures were repeated 11 times with our patch attached or wearing a commercial device using the developed recording program (Figure S6, Supporting Information). The first trial was used as the training data set for training the machine‐learning classifier, and the remaining ten trials were used as the test dataset. As summarized in Figure 5E, the classification accuracies of the SED and the commercial Myo are 95.11 ± 9.81% and 89.71 ± 6.08%, respectively. The SED performs better than the commercial one regarding long‐term usability (*p*‐value = 0.0977). About 50 hand gestures have been classified with 100% accuracy. However, starting from the sixth trial, a subject expressed muscle fatigue in repeating the same gestures. The system would perform better practically by implementing a classification method that considers fatigue. Unlike our soft patch, the accuracy of the commercial device continued to decrease even from the first trial without muscle fatigue. Furthermore, from the sixth trial to the ninth trial, rapid fluctuation in performance occurred. For the tangible real‐world application of gesture classification, addressing these performance inconsistencies is imperative. As a method for preventing performance reduction caused by muscle fatigue and rapid changes in performance, domain adaptation techniques can be used. This method can adapt features and classifiers when data distributions differ.^[^ ^29^ ^]^ Because muscle fatigue and inconsistent signal quality cause changes in EMG signal patterns, that is, changes in data distribution, utilizing domain adaptation techniques can enhance the system's robustness, ensuring consistent performance despite muscle fatigue and signal variations in real‐life applications.

### 2.6 Demonstration of Real‐Time Continuous Control of an FPV Drone Using an AR‐Integrated Soft Electronic System

**Figure 6:**
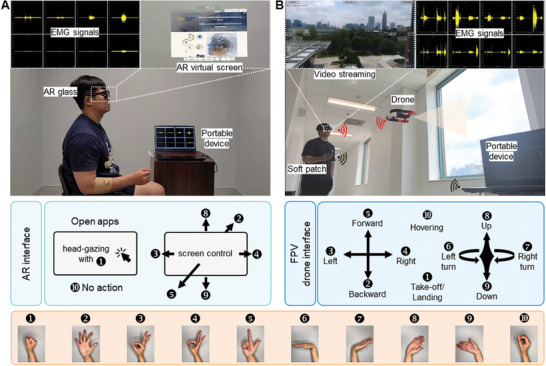
Demonstration of real‐time continuous control of an FPV drone using an AR‐integrated soft electronic system. A) Overview of an AR interface that a subject interacts, capturing multi‐channel EMG signals from the wearable patch on the forearm. Nine different gestures shown below control the AR interface. B) Real‐time control of an FPV drone using AR‐integrated electronics. Ten actions of drone control include take‐off/land, moving left, right, forward, backward, up, down, left turn, right turn, and hovering.

In this study, we demonstrate the performance of an AR‐integrated SED for wireless, real‐time, continuous control of an FPV drone (**Figure** **6**). The AR interface, built on the SED, opens new horizons for enhancing productivity and collaboration within screen‐based working environments. AR can transform complex data into manipulable 3D graphs or charts, offering intuitive insights directly through the screen.^[^ ^30^ ^]^ It also plays a vital role in accessibility, molding screen content to cater to the specific needs and preferences of individuals with disabilities.^[^ ^31^ ^]^ An overview of the implemented AR interface is explained in Figure 6A, showing a subject wearing the SED and AR glasses (Nreal Light) with eight‐channel EMG signals, and an example of an AR virtual screen. Using the “fist” gesture, a subject can open a virtual screen with a mouse click. Then, the window's position can be adjusted as needed. There are ten different gestures used to control this AR interface (details of an example in Video S1, Supporting Information). Based on the developed AR interface, we utilized the wearable SED to control an FPV drone with EMG signals. Using goggles or headsets that confine the user to the screen while operating a machine, such as a drone, can completely immerse the user in a screen and the operation of the machine. However, this total immersion can be a disadvantage when operating machines in industrial or hazardous environments where real‐world interaction and awareness are essential, so it is appropriate to use AR that can be aware of their surroundings. AR glasses can guide the operator by displaying video and other telemetry data, such as altitude, speed, and battery status, directly in the drone pilots' field of view in real‐time. Figure 6B summarizes an example of controlling the drone with a camera for sending a video feed to external devices. Through the interfaces, a subject in Figure 6B could precisely control the drone using ten different gestures, including take‐off/land, moving left, right, forward, backward, up, down, left turn, right turn, and hovering. An example of drone control appears in Video S2 (Supporting Information). By synergizing AR, FPV drones, and our SED, we validate the proof of concept and underscore the system's expandability, illustrating its potential to revolutionize user interaction and control in diverse industrial applications without the need for additional hand‐held displays or controllers.

## 3 Conclusion

This paper reports on innovative AR and human‐machine interfaces controlled by EMG data from a scalable SED. The wearable device with an array of electrodes allows for skin conformality, long‐term wearability, multiple uses, and wireless data transfer for detecting various types of muscle activities with high accuracy. The SED shows more reliable and higher sensing performance than a commercial device. The Riemannian feature‐based classification developed here offers 96.08% accuracy in classifying ten hand gestures with only 1‐min training. The demonstration of real‐time continuous control of an FPV drone captures the capabilities of the AR‐integrated SED with eight‐channel EMG electrodes. In this example, a user could utilize ten gestures as virtual screen control commands for drone teleoperation. The SED's signal quality and classification performance can be affected by physiological characteristics, such as skin roughness, muscle mass, and fat mass on the forearm. Therefore, for the practical applications of the SED in the fields of industry, agriculture, and military, it would be a promising topic to investigate the influence of various physiological factors on the performance of the SED, enhancing its adaptability and efficiency across diverse user profiles. Furthermore, hand gesture recognition technology presented in this work can play an essential role in various applications, such as prosthetic control for amputees, surgeon control of robotic‐assisted systems, and sign language recognition for deaf people. Future work would focus on adding densely packed electrodes and detecting additional motions for persistent human–machine interfaces.

## 4 Experimental Section

### Skin Contact Impedance

To measure the skin‐electrode contact impedance, two electrodes were connected to a skin impedance meter (model 1089NP Checktrode, UFI). Before attaching electrodes, the skin was properly cleaned with skin preparation gel (NuPrep Skin Prep Gel, Weaver and Company). Then, medical tape attached two serpentine electrodes to the forearm (Tegaderm, 3 M).

### Computational Simulation

The SED's mechanical deformations and strain distributions were characterized using 3D FEA. This study was performed using the commercial software ABAQUS to find out an ideal design for the device.

### Experimental Validation of Electro‐Mechanical Reliability

For validating the electro‐mechanical reliability of SED, samples of forearm patches were mechanically bent using a motorized testing machine (ESM303, Mark‐10) with a speed of 115 mm min^−1^. For the cyclic bending test, the sample was repeatedly bent and unfolded at the same speed for 100 cycles (about 3.5 h). In addition, samples of serpentine electrodes were mechanically stretched using ESM303 with a speed of 15 mm min^−1^. For the cyclic stretching test of the electrode, the samples were repeatedly stretched and relaxed at 15 mm min^−1^ speed for 100 cycles (≈1 h).

### Study of the Peeling Strength of Adhesive Substrate

Three test samples of four different adhesive substrates (9907T+S, 9907T, 2476P, and 2480) were prepared in 3.81 x 10.16 cm (1.5 x 4‐inch). “9907T+S” indicates the adhesive substrate that the Silbione (A‐4717, Factor II Inc.) was coated on the 9907T. To measure the peeling strength of each sample, it was attached to the forearm after properly cleaning the skin using an alcohol swipe. And the sample was mechanically peeled vertically with a motorized force tester (ESM303, Mark‐10) at a 30 mm min^−1^ speed. The force tester recorded adhesion force data during the test until full detachment. To quantify the peeling energy, the area under the force‐distance curves (Figure S4, Supporting Information) was calculated using the “trapz” function of MATLAB and divided by the substrate area (3.81 x 8 cm). To measure the peeling energy of adhesive substrates on the wet skin, 2 mL of water was dropped on the skin before attaching samples to the skin.

### Statistical Analysis and Feature Visualization

All statistical analyses were performed using MATLAB R2021b (Mathworks, Natick, MA, USA). A parametric or nonparametric statistical test was selected based on the result of the Kolmogorov–Smirnov test, which tests the Gaussianity of a dataset.^[^ ^32^ ^]^ When the difference between the two sets was tested, a paired *t*‐test or Wilcoxon signed rank test was selectively used. The t‐SNE function was used in MATLAB R2021b to visualize the distribution of the extracted Riemannian feature. The algorithm of this function was selected as “exact”, and the other parameters were set to default.

### Human Subject Study

Five healthy volunteers (male: 5, age: 27.8 ± 4.49 years, forearm circumference: 27.53 ± 1.82 cm) participated in this experiment. During the experiment, subjects were asked to make hand/wrist gestures following the instructions (Figure S6, Supporting Information). Each subject participated in the experiment three times and repeated ten gestures three times in one experiment. The participants had no history of neurological, psychiatric, or other severe diseases that could affect the experimental results. The Georgia Tech IRB approved the experimental protocol (#20 226). Each participant was informed of the detailed experimental protocol before participating.

## Conflict of Interest

Georgia Tech has a pending patent application related to the work described here.

## Author Contributions

H.K. and H.‐S.C. contributed equally to this work. H.K., H.‐S.C., C.‐H.I., and W.‐H.Y. designed the research. H.K., H.‐S.C., M.K., Y.J.L., S.H.L., S.I., H.K., C.‐H.I., and W.‐H.Y. performed the research. H.K., H.‐S.C., M.K., H.Y., and W.‐H.Y. analyzed the data. H.K., H.‐S.C., M.K., Y.J.L., and W.‐H.Y. wrote the paper.

## Supporting information

Supporting Information

Supplemental Video 1

Supplemental Video 2

## Data Availability

The data that support the findings of this study are available from the corresponding author upon reasonable request.
