# Supplementary material for: AR‐Enabled Persistent Human–Machine Interfaces via a Scalable Soft Electrode Array
Source: Adv Sci (Weinh). 2023 Dec 13;11(7):2305871. doi: 10.1002/advs.202305871 (PMC10870043; doi:10.1002/advs.202305871)
Supplement: Supplementary file 1 — Supporting Information [file ADVS-11-2305871-s001.pdf]

ADVANCED SCIENCE
Open Access

Supporting Information

for *Adv. Sci.*, DOI 10.1002/advs.202305871

AR-Enabled Persistent Human–Machine Interfaces via a Scalable Soft Electrode Array

Hodam Kim, Ho-Seung Cha, Minseon Kim, Yoon Jae Lee, Hoon Yi, Sung Hoon Lee, Soltis Ira, Hojoong Kim, Chang-Hwan Im and Woon-Hong Yeo**

Supporting Information

AR-Enabled Persistent Human-Machine Interfaces via a Scalable Soft Electrode Array

Hodam Kim, Ho-Seung Cha, Minseon Kim, Yoon Jae Lee, Hoon Yi, Sung Hoon Lee, Soltis Ira, Hojoong Kim, Chang-Hwan Im and Woon-Hong Yeo**

Dr. H. Kim, Dr. H.-S. Cha, Y. J. Lee, Dr. H. Yi, S. H. Lee, S. Ira, Dr. H. Kim, and Prof. W.-H. Yeo
IEN Center for Human-Centric Interfaces and Engineering, Institute for Electronics and Nanotechnology, Georgia Institute of Technology, Atlanta, GA 30332, USA
Dr. H. Kim, Dr. H.-S. Cha, Dr. H. Yi, S. Ira, Dr. H. Kim, and Prof. W.-H. Yeo
George W. Woodruff School of Mechanical Engineering, College of Engineering, Georgia Institute of Technology, Atlanta, GA 30332, USA
Dr. H.-S. Cha and Prof. C.-H. Im
Department of Biomedical Engineering, Hanyang University, Seoul, 04763, Republic of Korea
E-mail: ich@hanyang.ac.kr (C.-H. Im)
M. Kim
School of Mechanical Engineering, Soongsil University, 369 Sangdo-ro, Dongjak-gu, Seoul 06978, Republic of Korea
Y. J. Lee and S. H. Lee
School of Electrical and Computer Engineering, College of Engineering, Georgia Institute of Technology, Atlanta, GA 30332, USA
Prof. W.-H. Yeo
Wallace H. Coulter Department of Biomedical Engineering, Parker H. Petit Institute for Bioengineering and Biosciences, Institute for Materials, Institute for Robotics and Intelligent Machines, Neural Engineering Center, Georgia Institute of Technology, Atlanta, GA 30332, USA
E-mail: whyeo@gatech.edu (W.-H. Yeo)

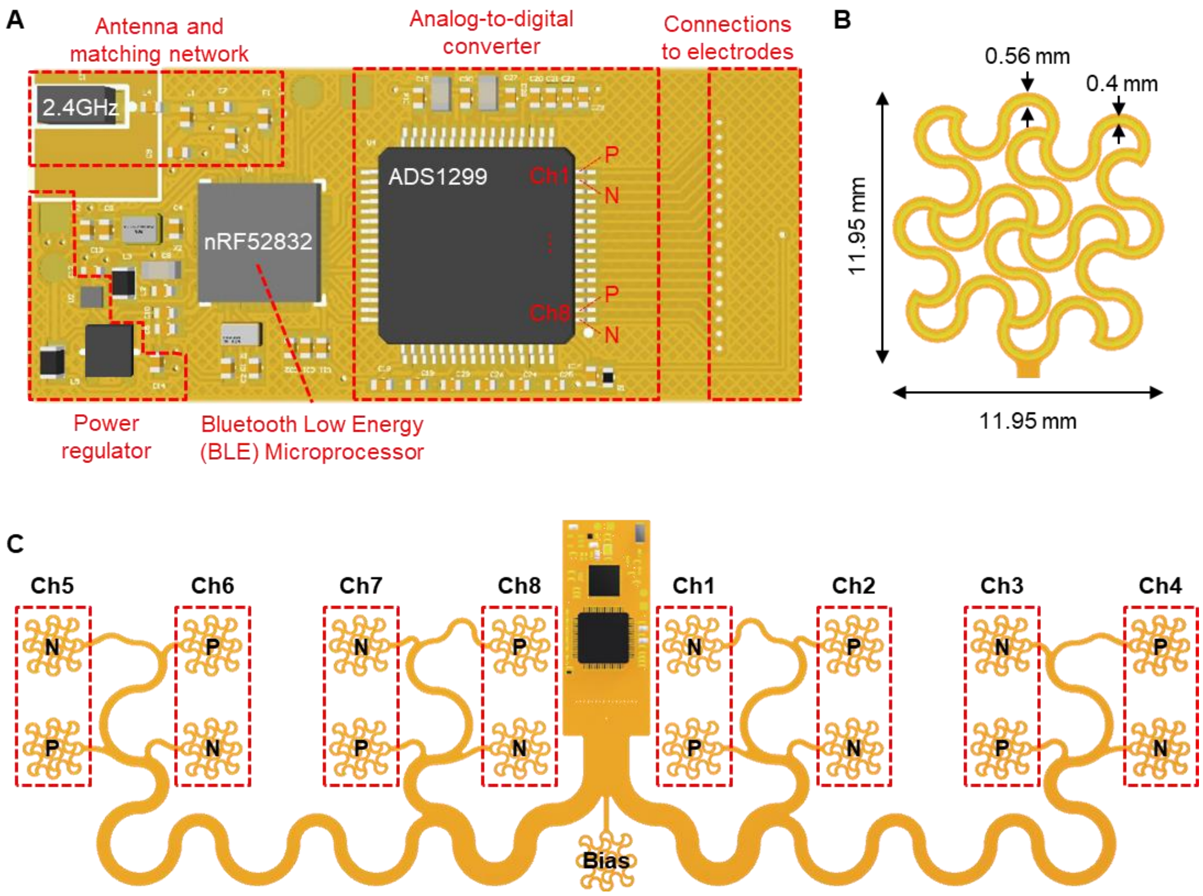

Figure S1. Circuit and electrode design. (A) Top-view illustration of circuit component with highlighted function blocks. (B) Bottom-view illustration of an electrode. (C) Schematic illustration of electrode array

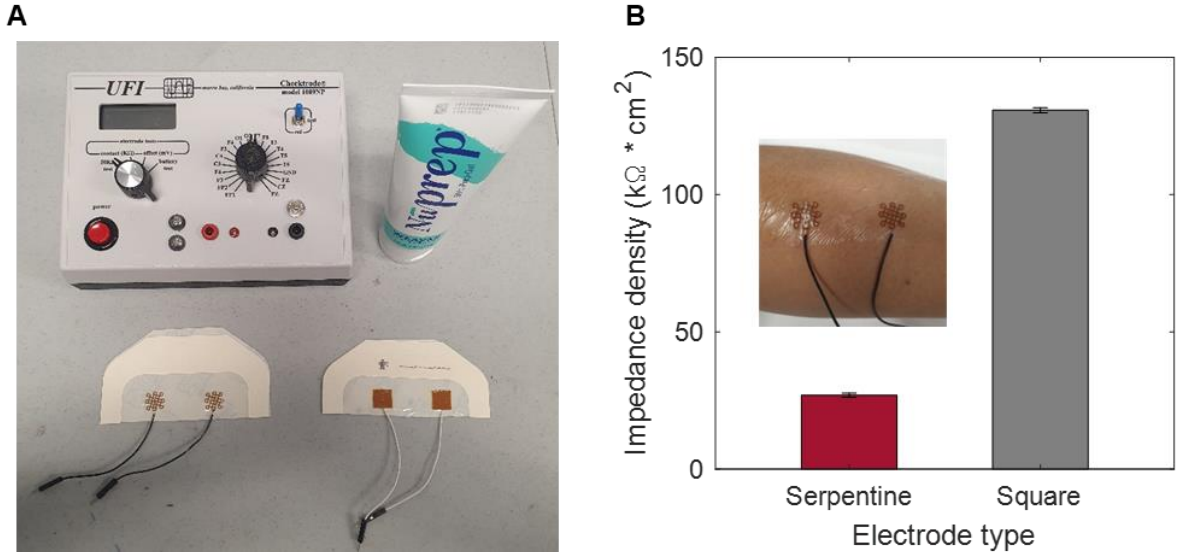

Figure S2. Skin-electrode contact impedance density. (A) Experimental setting. (B) Bar graphs of skin-electrode contact impedance density of two types of electrodes.

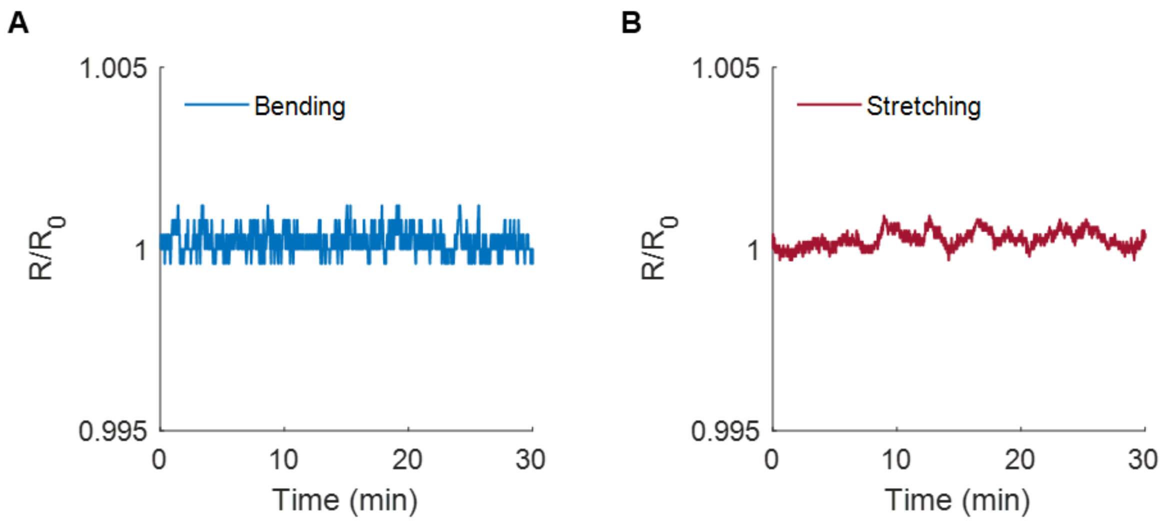

Figure S3. (A) Measured electrical resistance of the interconnector and electrodes during continuous bending strain (bending radius: 45mm). (B) Electrical resistance of an electrode during continuous stretching strain (strain: 15%)

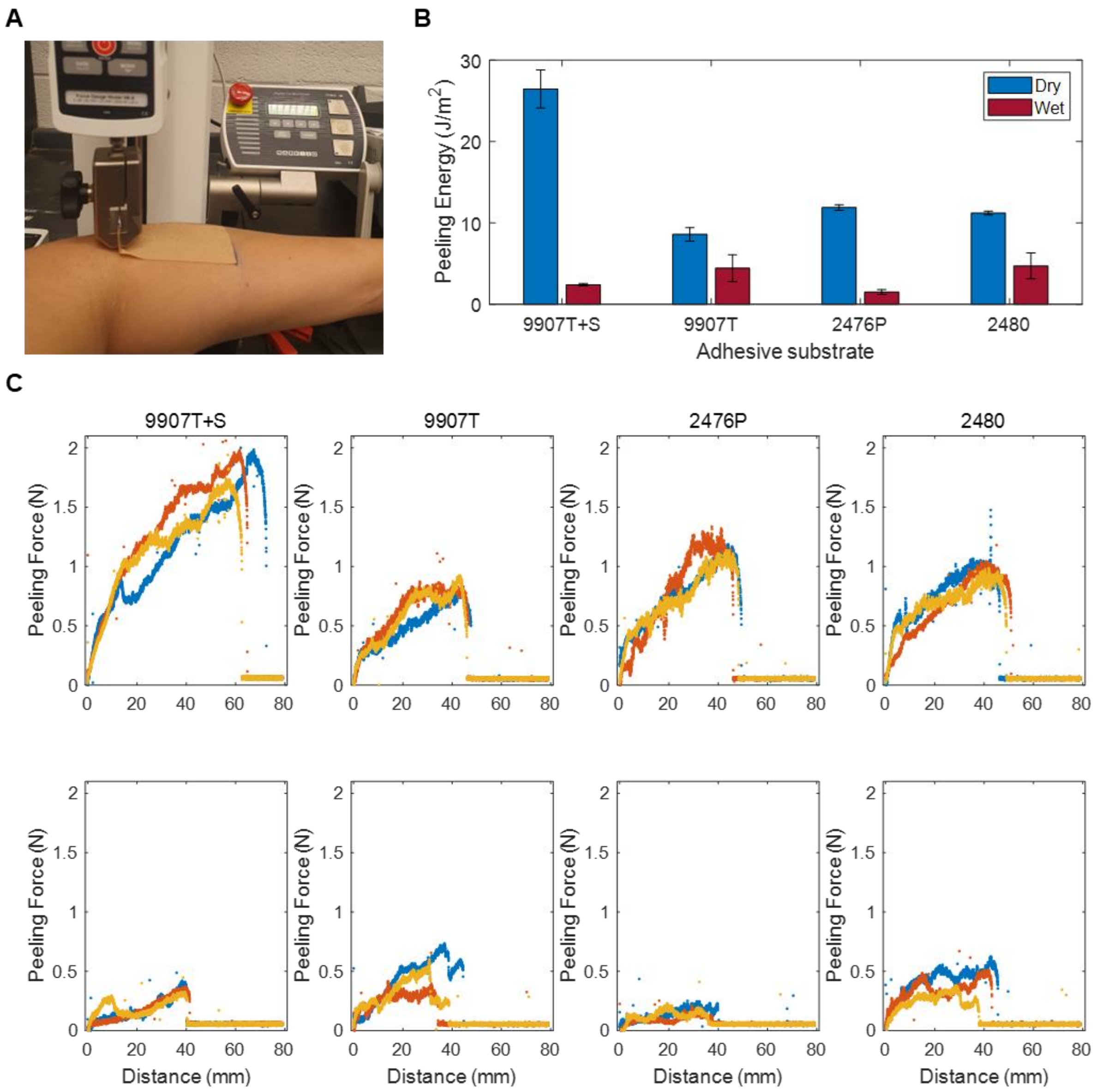

Figure S4. Experimental setup and result of peeling test (A) Photo of experimental setup for testing peeling strength. (B) Peeling energy of four adhesive substrates in the dry and wet skin conditions (C) Distance-Peeling force curve for each substrate and each skin conditions.

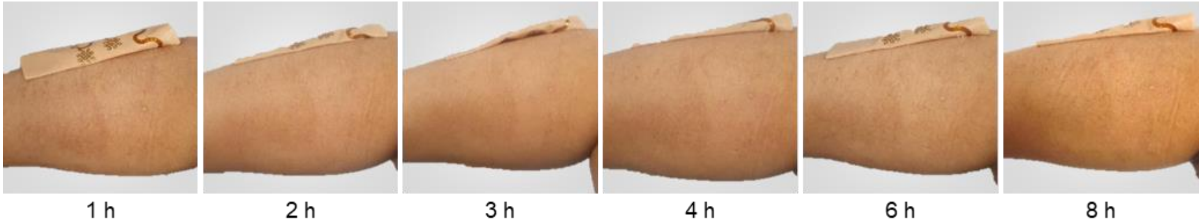

Figure S5. Photos showing changes in skin while wearing a soft electronic device on the forearm. No side effects were shown to the skin after wearing over 8 hours.

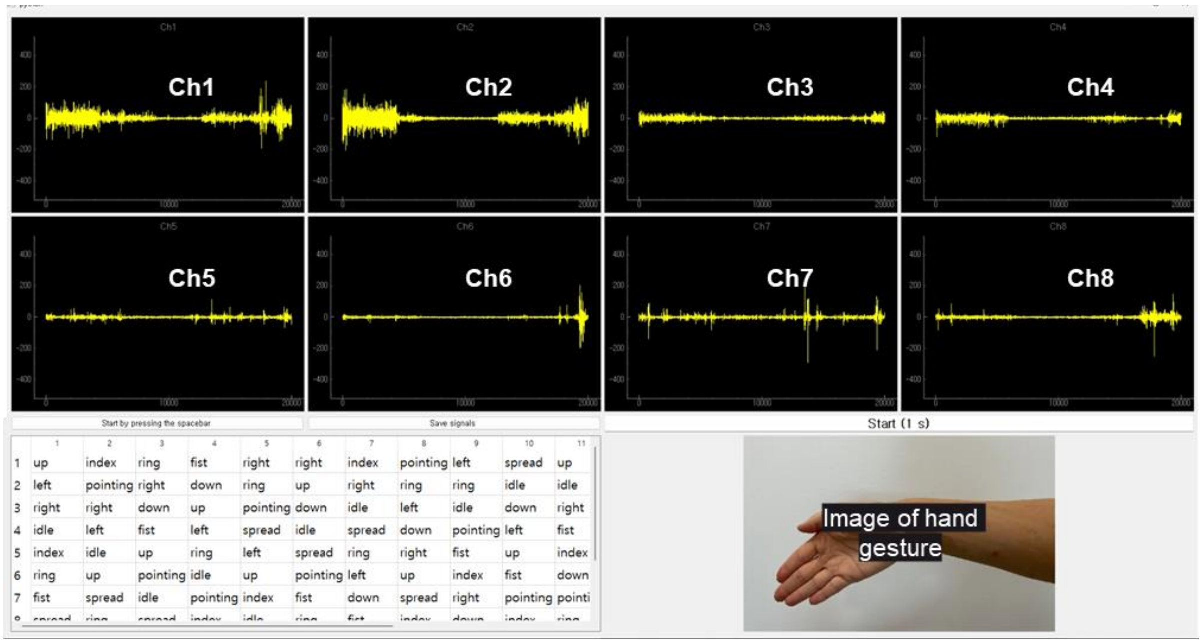

Figure S6. Developed program for recording EMG signals and presenting hand gesture images to be performed by the subject.

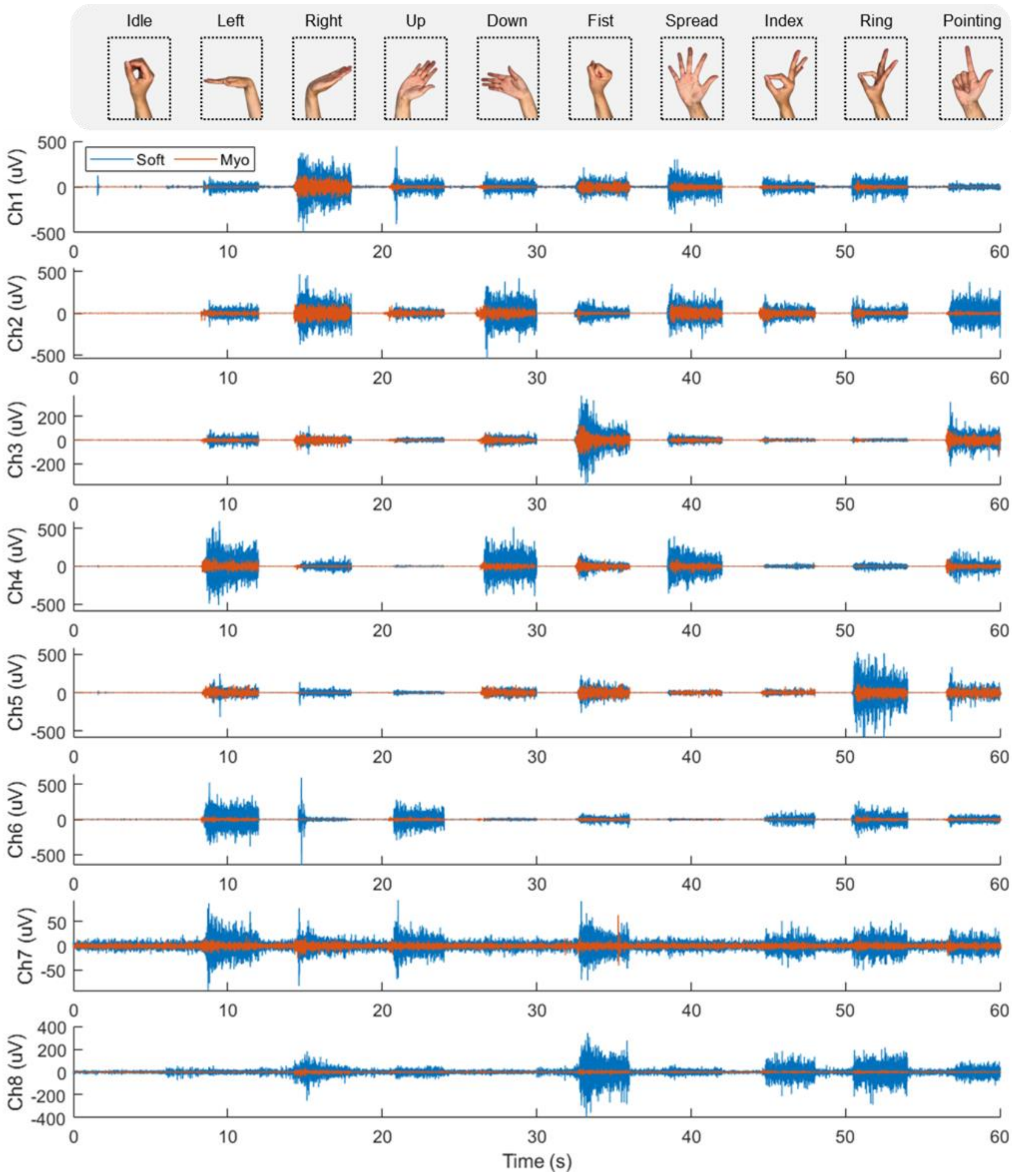

Figure S7. Representative EMG signals measured by a soft wearable device and a commercial device (Myo) with eight channels when a subject makes ten different gestures.

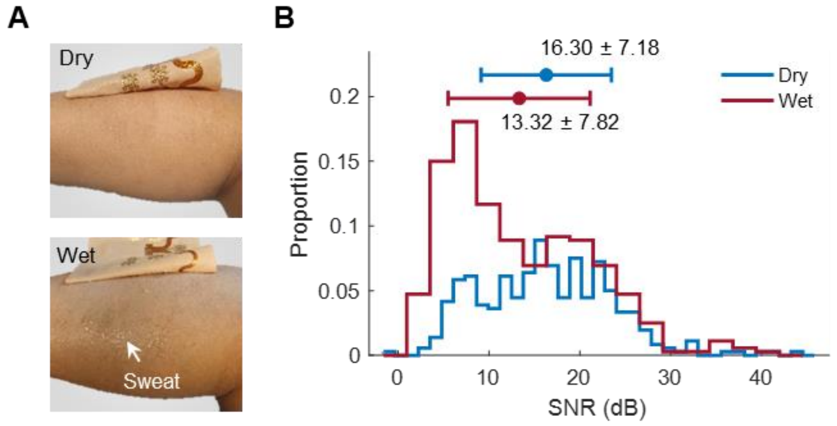

Figure S8. Comparison of performance under dry and wet skin conditions. (A) Photos of an experiment for testing performance. (B) Comparison of signal-to-noise ratio (SNR) of two skin condition.

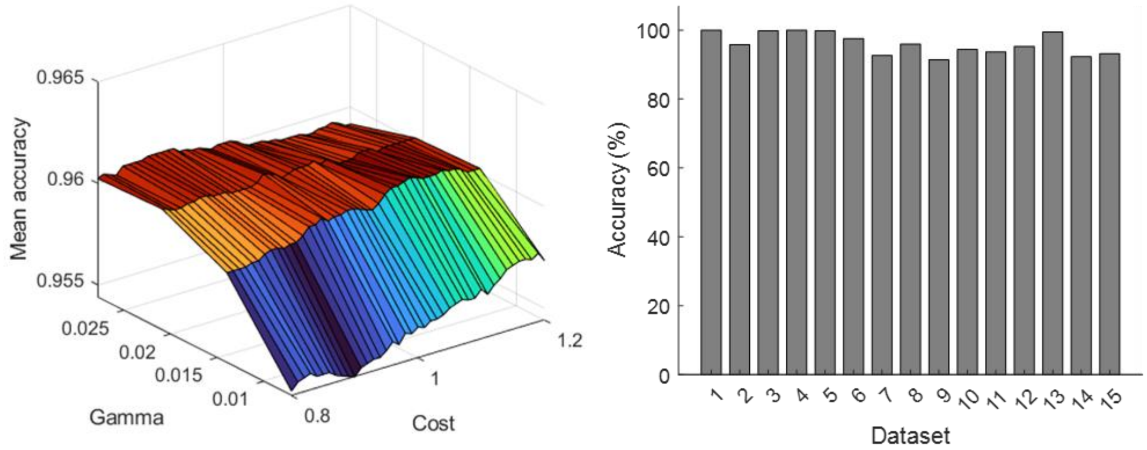

Figure S9. Result of gird search for the hyperparameter (Cost: 0.8 to 1.2; Gamma: 1/144 to 1/36) optimization and the classification accuracy of each dataset.

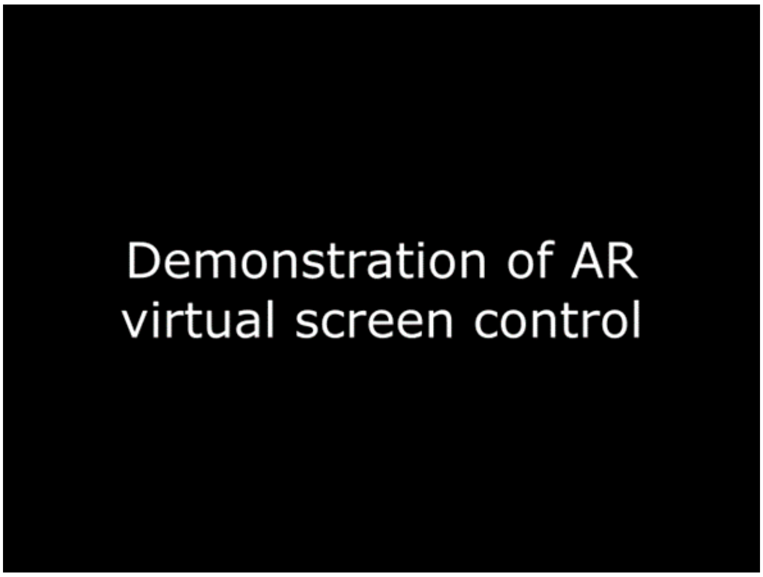

Video S1. Demonstration video of AR virtual screen using a soft patch.

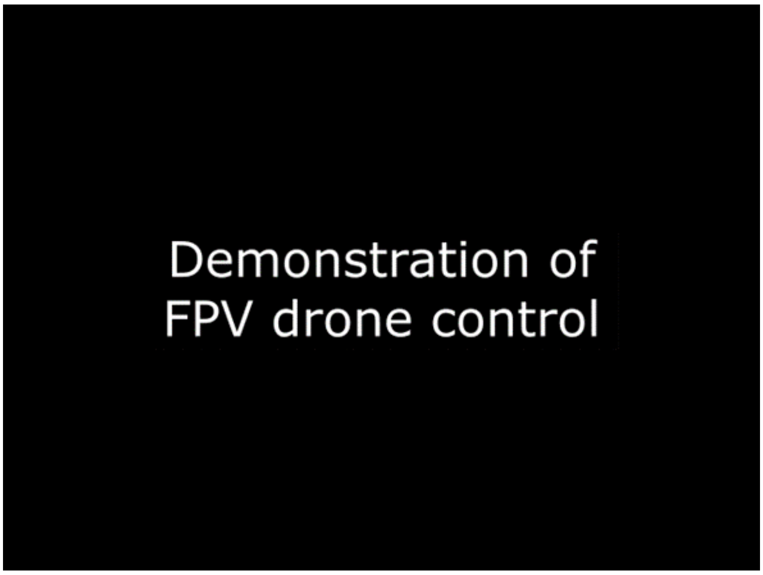

Video S2. Demonstration video of FPV drone control using a soft patch.
